# Supplementary material for: FSHβ links photoperiodic signaling to seasonal reproduction in Japanese quail
Source: eLife. 2023 Dec 27;12:RP87751. doi: 10.7554/eLife.87751 (PMC10752586; doi:10.7554/eLife.87751)
Supplement: Supplementary file 1. — Short photoperiods activate an endogenously generated programmed to increase follicle-stimulating hormone-β (FSHβ) expression in the pars distalis of the pituitary gland. A gradual increase in non-stimulatory photoperiods, such as 10v and 12v, establish the photosensitive state and characterized by constituently expressed FSHβ. Photoperiods that extend beyond the critical daylength (e.g., >12v) activates thyrotropes thyrotropin-stimulating hormone-β (TSHβ) expression in the pars tuberalis of the pituitary gland. The coincidence timing of long day TSHβ with the short day photosensitivity induced by increased FSHβ expression results in gonadal development. Figure was created using BioRender. [file elife-87751-supp1.pdf]

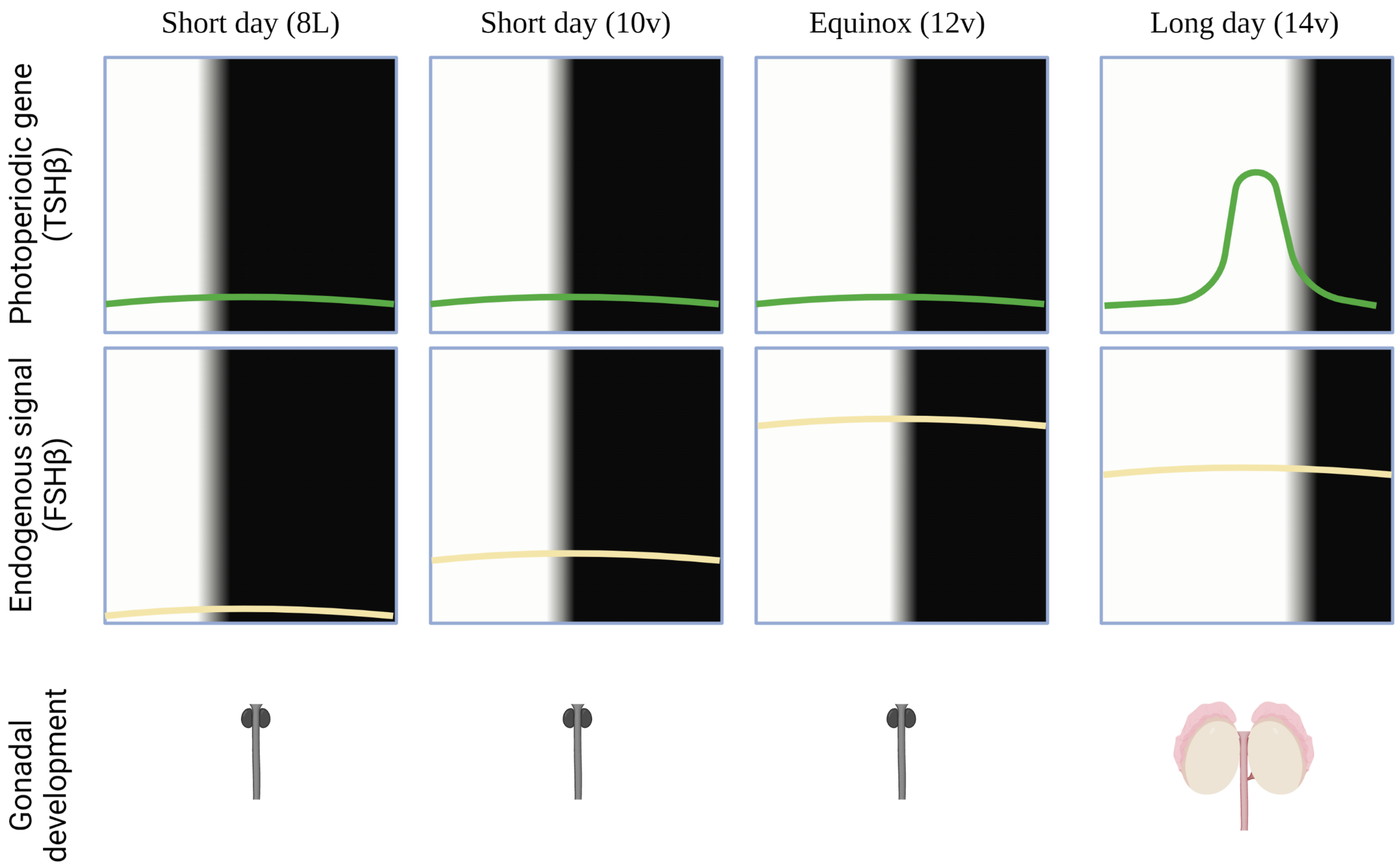

Two distinct pituitary cell types are involved in the external coincidence model for the avian photoperiodic response. Short photoperiods activate an endogenously generated programmed to increase *FSHβ* expression in the pars distalis of the pituitary gland. A gradual increase in non-stimulatory photoperiods, such as 10v and 12v, establish the photosensitive state and characterised by constituently expressed *FSHβ*. Photoperiods that extend beyond the critical day length (e.g., >12v) activates thyrotropes *TSHβ* expression in the pars tuberalis of the pituitary gland. The coincidence timing of long day *TSHβ* with the short day photosensitivity induced by increased *FSHβ* expression results in gonadal development. Figure was created using BioRender.
