# Supplementary figures and images for: FSHβ links photoperiodic signaling to seasonal reproduction in Japanese quail
Source: eLife. 2023 Dec 27;12:RP87751. doi: 10.7554/eLife.87751 (PMC10752586; doi:10.7554/eLife.87751)

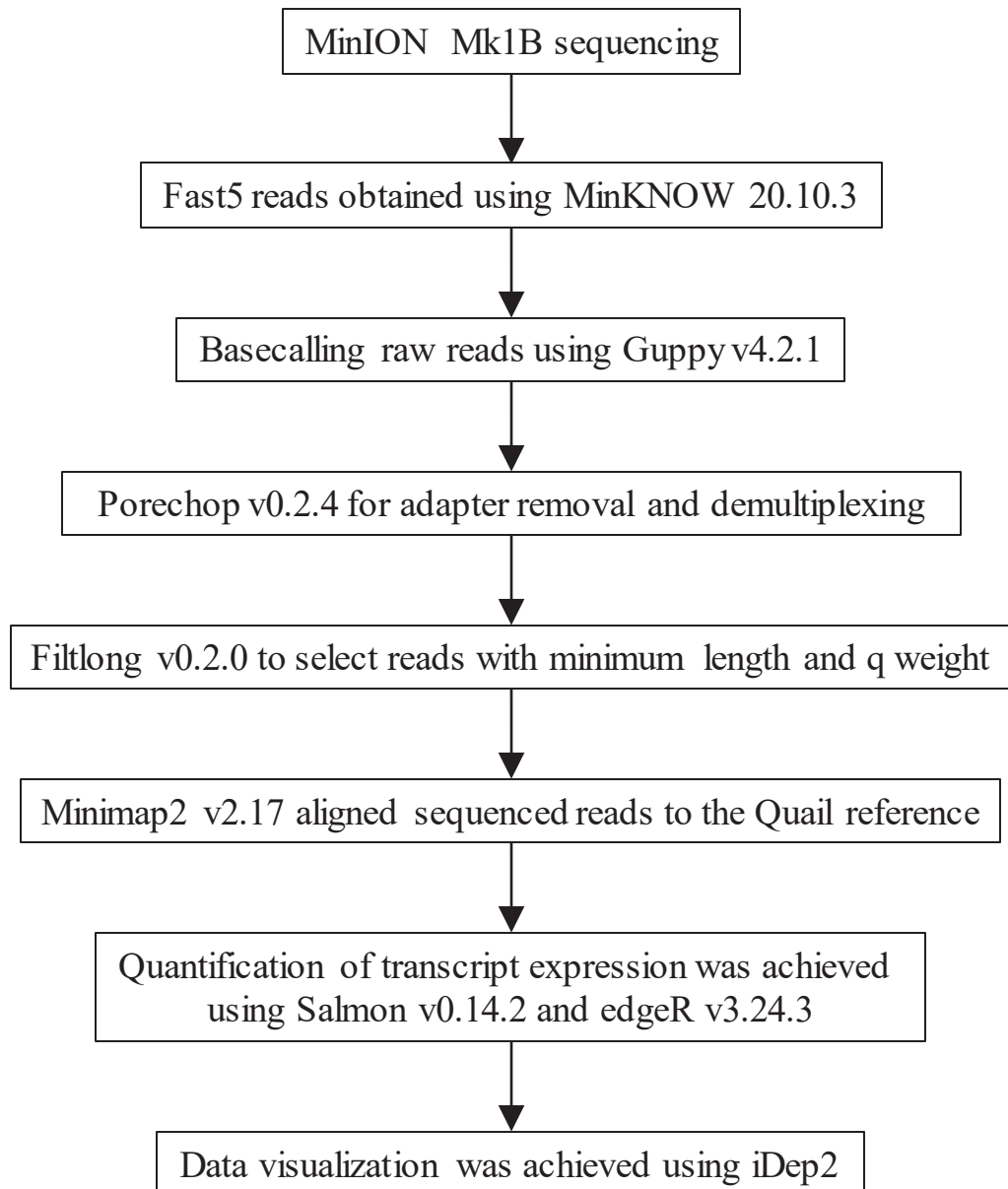

Supplement: Supplementary file 3. [file elife-87751-supp3.pdf]
